# TASK-3 Downregulation Triggers Cellular Senescence and Growth Inhibition in Breast Cancer Cell Lines

**DOI:** 10.3390/ijms19041033

**Published:** 2018-03-29

**Authors:** Rafael Zúñiga, Claudio Valenzuela, Guierdy Concha, Nelson Brown, Leandro Zúñiga

**Affiliations:** Centro de Investigaciones Médicas (CIM), Programa de Investigación Asociativa en Cáncer Gástrico (PIA-CG), Escuela de Medicina, Universidad de Talca, Talca 3460000, Chile; rafaelzunigah@gmail.com (R.Z.); clavalenzu@gmail.com (C.V.); guierdy@gmail.com (G.C.); nbrown@utalca.cl (N.B.)

**Keywords:** TASK-3, two-pore domain channel, breast cancer, cell cycle, senescence, cyclin-dependent kinase inhibitors

## Abstract

TASK-3 potassium channels are believed to promote proliferation and survival of cancer cells, in part, by augmenting their resistance to both hypoxia and serum deprivation. While overexpression of TASK-3 is frequently observed in cancers, the understanding of its role and regulation during tumorigenesis remains incomplete. Here, we evaluated the effect of reducing the expression of TASK-3 in MDA-MB-231 and MCF-10F human mammary epithelial cell lines through small hairpin RNA (shRNA)-mediated knockdown. Our results show that knocking down TASK-3 in fully transformed MDA-MB-231 cells reduces proliferation, which was accompanied by an induction of cellular senescence and cell cycle arrest, with an upregulation of cyclin-dependent kinase (CDK) inhibitors p21 and p27. In non-tumorigenic MCF-10F cells, however, TASK-3 downregulation did not lead to senescence induction, although cell proliferation was impaired and an upregulation of CDK inhibitors was also evident. Our observations implicate TASK-3 as a critical factor in cell cycle progression and corroborate its potential as a therapeutic target in breast cancer treatment.

## 1. Introduction

Breast cancer is one of the most prevalent types of cancer affecting women [1] and remains a leading cause of cancer-related mortality worldwide [2]. In spite of their common tissue of origin, breast tumors display an extensive heterogeneity, which is reflected by a diverse array of molecular and histological subtypes [3]. Despite important therapeutic advances in chemotherapy and adjuvant endocrine therapies [4], a number of breast cancers are likely to require the development of new therapeutic approaches in order to confront the almost certain development of drug resistance.

Over the last years, accumulating evidence has supported the involvement of potassium (K^+^) channels in cellular processes commonly disrupted in cancer [5,6]. Indeed, several reports have associated the expression and function of K^+^ channels with cancer progression, making these channels attractive targets for novel cancer therapies and useful diagnostic tools [7,8]. K^+^ channels have been associated to several hallmarks of cancer, such as sustained proliferation, migration, invasion, angiogenesis, and metastasis [6,9,10,11,12,13,14].

Mechanistically, K+ channels selectively transport K^+^ ions across cell membranes and play a crucial role in maintaining resting membrane potentials in various cell types [15,16]. K^+^ channel-regulated cell membrane potential is also essential during cell cycle progression [17], as well as in the regulation of cell death by necrosis and apoptosis [12,18].

Recent studies have begun to unveil the contribution of two-pore domain (K2P) K^+^ channels to the establishment of some of the hallmarks of cancer [6,10,19,20]. So far, fifteen members of the K2P family have been identified. This family can be divided into six subfamilies denoted as TREK, TALK, TASK, TWIK, THIK, and TRESK [21,22,23]. Of all K2P channel family members, four have been involved in cancer (TASK-1, -2, -3 and TREK-1) [12,24,25]. Among these, TASK-3 (also known as K2P9.1), encoded by the *KCNK9* gene, has been recognized for its potential oncogenic properties [26]. TASK-3 is highly expressed in neurons of the central nervous system, including the cerebellum [15,16,27,28], where it contributes to generate resting and action potentials [15,16,29]. Importantly, *KCNK9* can be overexpressed in up to 44% and 35% of human breast and lung tumors, respectively [30]. Additionally, *KCNK9* has been reported to be overexpressed in over 90% of ovarian tumors [31]. More recently, overexpression of this channel at the protein level has been documented in colorectal cancer and melanoma [18,31,32]. Of note, heterologous overexpression of TASK-3 has been shown to induce tumorigenesis in experimental animal models, confirming its oncogenic properties [10].

Gain of function of TASK-3 is associated with the acquisition of several malignant characteristics, including resistance to hypoxia and serum deprivation [30]. Recently, it has been shown that the use of monoclonal antibodies against the cap domain of TASK-3 inhibits tumor growth and metastasis in animal models with no significant side effects [33,34].

Here we examine the expression of TASK-3 in the triple-negative (ER, PR, and HER-2 negative) breast cancer cell line MDA-MB-231, a cell line that is also deficient in the p53 suppressor gene [35], and in the non-transformed human breast cancer cell line MCF-10F. From a clinical standpoint, triple negative breast cancer cells are more aggressive and metastatic, commonly failing to respond to current pharmacological approaches (such as Herceptin and Estrogen antagonists). Therefore, the development of more effective therapies to treat these tumors remains a challenge. Our results show that knocking down TASK-3 leads to reduced proliferation in MDA-MB-231 cells and identified cellular senescence as the likely mechanism involved. In addition, TASK-3 downregulation also reduced proliferation in the non-tumorigenic cell line MCF-10F, although we were unable to document signs of permanent cell cycle arrest (senescence).

## 2. Results

### 2.1. Expression of TASK-3 Channels in MDA-MB-231 and MCF-10F Cells

**Figure 1:**
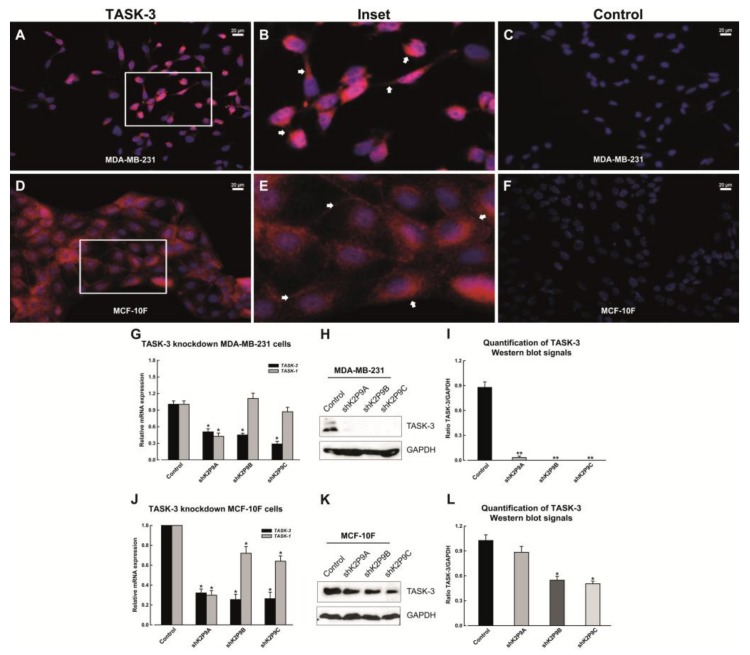
Immunofluorescence and expression analyses of TASK-3 in MDA-MB-231 and MCF-10F cell lines. (**A**,**B**,**D**,**E**) Immunofluorescence localization of TASK-3 channel (red fluorescence); (**B**,**E**) inset showing a magnification of the indicated area. White arrows indicate examples of membrane localization of TASK-3; (**C**,**F**) immunostaining when the primary antibodies were omitted (control). DAPI was used for nuclear staining (blue fluorescence). The scale bar represents 20 µm; (**G**,**J**) expression of TASK-3 (*KCNK9*) and TASK-1 (*KCNK3*) genes in cells transduced with either pMKO.1 empty vector (control) or shRNAs directed against TASK-3 (shK2P9A, shK2P9B, and shK2P9C) was assessed by quantitative real-time PCR. Gene expression was normalized against Homo sapiens ribosomal protein L19 (RPL19) using the ΔΔ*C*_t_ method. Error bars correspond to mean ± SEM (*n* = 3); (**H**,**K**) western blot analysis for TASK-3 detection following shRNA-mediated knockdown of TASK-3. Representative immunoblots for TASK-3 and GAPDH are shown. (**I**,**L**) The relative abundance of TASK-3 is expressed as the ratio between the intensity of the TASK-3 band of treated samples and the control sample, normalized on intensity of the GAPDH band (loading control). Data are expressed as mean ± SEM of three independent experiments. For (**G**,**I**,**J**,**L**) * *p* < 0.05, compared with the control, based on one-way ANOVA with Tukey HSD (Honestly Significant Difference) post-test.

We first examined the expression of TASK-3 by immunofluorescence in tumorigenic MDA-MB-231, as well as in non-tumorigenic MCF-10F cells. Positive staining for TASK-3 was detected in both types of cells (Figure 1A,B,D,E) with an expected membrane localization pattern (arrows, Figure 1B,E). This result indicates that TASK-3 channel is stably expressed on the surface of both tumorigenic and non-tumorigenic mammary epithelial cell lines. The positive signal was not detected when the primary antibody was omitted (control, Figure 1C,F). In order to corroborate the immunofluorescence results, TASK-3 mRNA expression was determined by quantitative real-time PCR. In agreement with the immunofluorescence results, TASK-3 was also detectable at the mRNA level in both cell lines, although expression was clearly higher in MCF-10F cells (Supplementary Figure S1).

### 2.2. Short Hairpin RNA-Mediated Knockdown of TASK-3

In order to study the effects of reducing the expression of TASK-3 in mammary epithelial cells, shRNA-mediated knockdown of TASK-3 was implemented and confirmed by both qPCR and Western blotting. MDA-MB-231 and MCF-10F cells were transduced with the vector control (pMKO.1) or three different shRNAs targeting TASK-3. As shown in Figure 1G,J TASK-3 mRNA levels (*KCNK9*) were significantly reduced in both cell lines following transduction with the shRNAs. The specificity of the knock down was confirmed by assessing the expression of TASK-1, a highly homologous TASK channel. As shown in Figure 1G,J, two shRNA constructs (shK2P9B and shK2P9C) displayed the highest specificity for TASK-3.

Next, TASK-3 knockdown was also evaluated by Western blotting (Figure 1H,K). A densitometric analysis of the relative intensity of the ~47 kDa TASK-3 band, after being normalized to GAPDH, is shown in Figure 1I,L. We observed a highly significant decrease in the levels of the TASK-3 protein in MDA-MB-231 cells subjected to shK2P9A-, shK2P9B- and shK2P9C-mediated knockdown compared to the levels of the protein in cells that that were transduced with the vector control (Figure 1I). In MCF-10F cells, however, the reduction in TASK-3 protein was only significant upon shK2P9B- and shK2P9C-mediated knockdown (Figure 1L). Given that the shK2P9B construct showed greater specificity for the TASK-3 channel, this construct was chosen for the next set of experiments.

### 2.3. Electrophysiological Characterization of TASK-3 Knockdown

**Figure 2:**
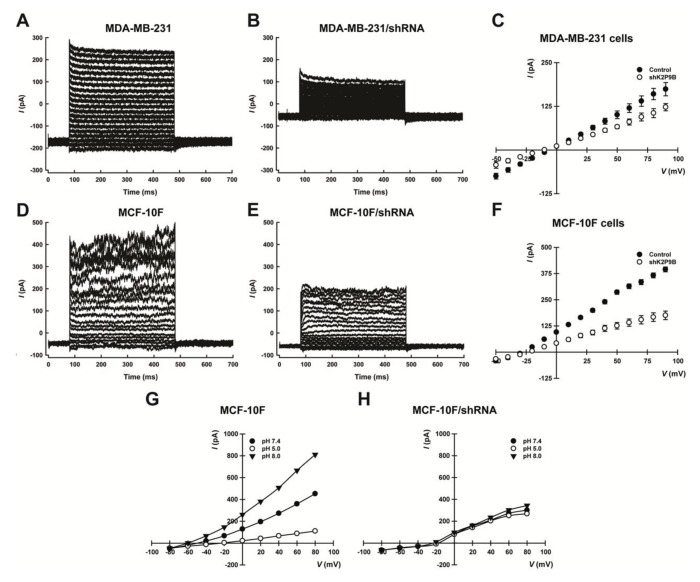
Whole-cell patch clamp measurements in cells with reduced expression of TASK-3. (**A**,**B**,**D**,**E**). Representative traces of potassium current were recorded in MDA-MB-231 and MCF-10F cells after transduction with either vector control (pMKO.1 puro) or shRNA directed against TASK-3 (shK2P9B); (**C**,**F**) current-voltage relationships determined in both conditions are shown; (**G**,**H**) I-V curves relationship of the pH-sensitive component was recorded at different extracellular pH solutions. Each MCF-10F cell was perfused with solution at pH 5.0 (open circle), pH 7.4 (open triangle), and pH 8.0 (closed circle).

We also evaluated the electrophysiological effects of knocking down TASK-3 in MDA-MB-231 and MCF-10F cells. To this end, the whole cell patch-clamp technique was used to record the macroscopic potassium current in MDA-MB-231 and MCF-10F cells that had been previously transduced with either the vector control (pMKO.1-puro) or an shRNA targeting TASK-3 (shK2P9B). We observed that reducing the expression of TASK-3 inhibited the macroscopic potassium current (Figure 2A,B,D,E). As shown in Figure 2C,F, the shRNA-mediated depletion of TASK-3 also decreased significantly the basal activity of TASK-3 channels in MDA-MB-231 and MCF-10F cells. Also, the extracellular pH changes, in a physiological range from pH 8.0 to 5.0, were used to demonstrate the contribution of TASK-3 (a pH-sensitive K2P channel) to the macroscopic potassium current. In agreement with previous studies [36,37], the macroscopic potassium current was strongly inhibited in the presence of an external pH of 5.0, compared to currents studied in the same cell at pH 7.4. In presence of an external alkaline pH (8.0), the opposite effect was observed (Figure 2G,H). As shown in Figure 2H, the shRNA-mediated depletion of TASK-3 also suppresses the pH-dependent potassium current in MCF-10F cells. These data clearly show that TASK-3 is a functional channel in both cell lines, and that the pH-dependent portion of the macroscopic potassium current is carried through TASK-3 channels.

### 2.4. Reducing the Expression of TASK-3 Inhibits Proliferation of MDA-MB-231 and MCF-10F Cells

**Figure 3:**
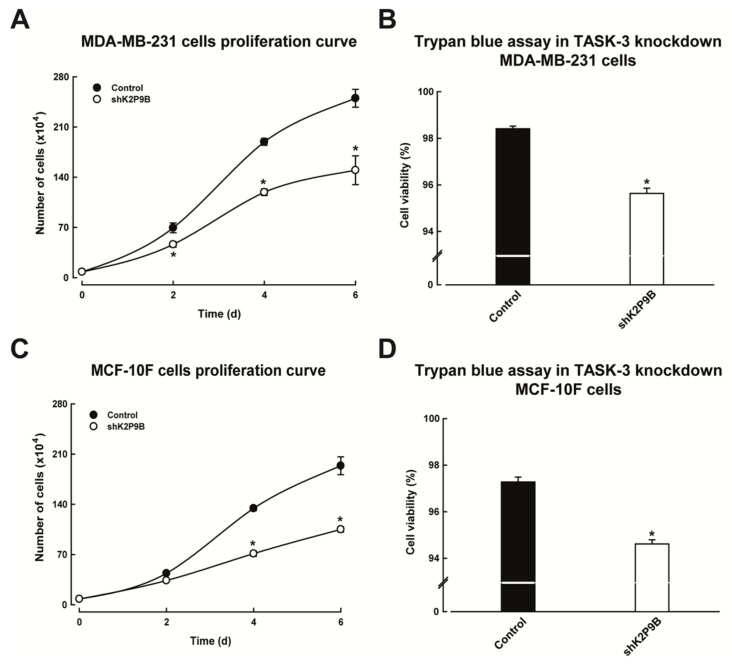
Effects of knocking down TASK-3 on cell proliferation and viability. (**A**,**C**) Proliferation curves of MDA-MB-231 and MCF-10F cells transduced with either pMKO.1 empty vector (control) or an shRNA targeting TASK-3 (shK2P9B). The values correspond to average ± SEM of four independent experiments. (*) indicates a significant difference between shRNA-treatment compared with the control (*p* < 0.05, *n* = 8 for each group, two-way ANOVA and Tukey’s multiple comparison test post); (**B**,**D**) viability was assessed using Trypan blue assay. Percentage of viability of MDA-MB-231 and MCF-10F cells after transduction with either vector control (pMKO.1 puro) or an shRNA against K2P9 (shK2P9B) is shown. Error bars represent the mean ± SEM of three independent experiments, each performed in triplicate. * *p* < 0.05, compared with the control, based on one-way ANOVA with Tukey’s post-test.

We next investigated the proliferative effects of knocking down TASK-3 in MDA-MB-231 and MCF-10F cells. To this end, cells previously transduced with either the pMKO.1-puro empty vector (control) or shRNAs targeting TASK-3 (shK2P9B) were counted after being propagated for 2, 4, and 6 days in culture. As shown in Figure 3A,C, the hairpin could reduce the accumulation of cells over time. A significant effect was evident from day 2, but it was most prominent on days 4 and 6 in MDA-MB-231 cells (Figure 3A). In MCF-10F cells a significant effect was observed on day 4, which remained significant up to day 6 (Figure 3C). These results indicate that shRNA-mediated depletion of TASK-3 reduces the accumulation of both tumorigenic and non-tumorigenic mammary epithelial cell lines, although it was not clear whether the effect was secondary to cell cycle arrest or an increase in cytotoxicity.

To distinguish between alternatives of cell fate, MDA-MB-231 and MCF-10F cells with reduced expression of TASK-3 were further evaluated for cell viability using the Trypan dye exclusion method. As shown in Figure 3B, MDA-MB-231 cells with reduced expression of TASK-3 displayed a marginal but statistically significant decrease in the percentage of viable cells compared to vector-transduced (control) cells (from 98.4 to 95.6%). The reduction in the viability of MCF-10F cells upon depletion of TASK-3 was also marginal but statistically significant (from 97.3 to 94.6%) (Figure 3D). These results indicate that reducing the expression of TASK-3 leads to a reduced accumulation of cells over time, and the major mechanism involved was not an increase in cytotoxicity.

In order to complement these studies, we also investigated the effects of hK2P9G201E, a dominant-negative mutant of TASK-3. The dominant-negative effect occurs in conditions where both the wild type and mutant subunits are co-expressed. In these conditions, the wild type subunits co-assemble with mutant subunits (carrying a site-directed mutation in the sequence encoding the pore region). Incorporation of these mutant subunits suppresses the functional properties of the channel. The dominant-negative mutant of TASK-3 was able to phenocopy the anti-proliferative effect observed in MDA-MB-231 cells with reduced expression of TASK-3 (Figure S2). A significant decrease in the number of cells was evident 48 h after hK2P9G201E transfection compared to cells transfected with an empty vector (Figure S2). The dominant negative effect of hK2P9G201E was also validated in HEK-293 cells (see Supplementary Figure S3). Thus, while HEK-293 cells expressing wild-type TASK-3 displayed electrophysiological properties concordant with leak potassium currents (Figure S3A,B), the co-expression of hK2P9G201E and wild type TASK-3 reduced the amplitude of TASK-3-associated currents (Figure S3C). These results indicate that the activity of TASK-3 as a conductor of background K^+^ currents is linked to the proliferative impairment observed in MDA-MB-231 cells with downregulation of TASK-3.

### 2.5. Cellular Senescence and Autophagy in TASK-3-Depleted MDA-MB-231 and MCF-10F Cells

**Figure 4:**
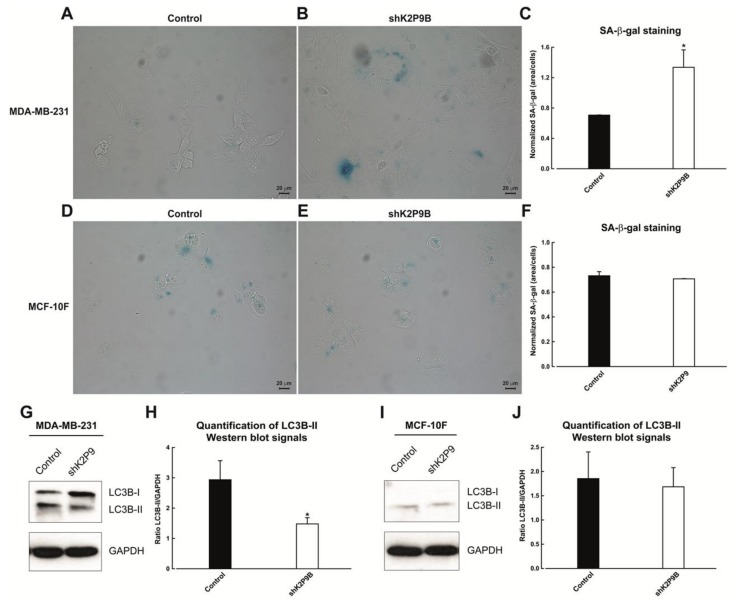
Cellular senescence and autophagy in TASK-3-depleted cell lines. (**A**,**B**,**D**,**E**) SA-β-gal activity in MDA-MB-231 and MCF-10F cells transduced with an shRNA targeting TASK-3 (shK2P9B) or vector control (pMKO.1). Representative bright-field images are shown; (**C**,**F**) quantification of the intensity of SA-β-gal staining by densitometric analyses; (**G**,**I**) immunoblot analysis of LC3B in TASK-3-depleted MDA-MB-231 and MCF-10F cells. Representative immunoblots for the detection of LC3B-I, LC3B-II and GAPDH in total cell lysates are shown. Quantitative densitometric analysis of blots are shown in (**H**,**J**), indicating the ratio between intensities of the LC3B-II and GAPDH bands in shRNA-treated and control samples. Data correspond to means ± SEM of three independent experiments. For (**C**,**F**,**H**,**J**) * *p* < 0.05, compared with the control, based on one-way ANOVA with Tukey HSD post-test.

To further understand the consequences of TASK-3 depletion in MDA-MB-231 and MCF-10F cells, cellular senescence was investigated. To this end, senescence-associated β-galactosidase (SA-β-gal) activity was assessed in cells that had been previously subjected to shRNA-mediated knockdown of TASK-3. The proportion of positive cells (blue cells) was determined by microscopic inspection (Figure 4A,B,D,E), and the intensity of SA-β-gal staining was quantified by densitometric analysis (Figure 4C,F). As shown in Figure 4C, shRNA-mediated knockdown of TASK-3 in MDA-MB-231 cells led to a significant increase in the proportion of SA-β-gal positive cells. These results indicate that knocking down TASK-3 inhibits cell proliferation through the induction of senescence in MDA-MB-231 breast cancer cells. In MCF-10F cells, however, shRNA-mediated knockdown of TASK-3 did no lead to significant changes in SA-β-gal activity (Figure 4F). These results suggest that knocking down TASK-3 inhibits cell proliferation through different mechanisms in MDA-MB-231 and MCF-10F cells.

We also assessed the status of autophagy in TASK-3-deficient MDA-MB-231 and MCF-10F cells, since this process has also been postulated as a mechanism of cell death in some experimental settings. To assess autophagy, the protein levels of the microtubule-associated protein 1 light chain 3 B (LC3B) were determined in cells with reduced expression of TASK-3 (Figure 4G,I). Following cleavage, LC3B becomes lipidated (LC3B-II) and localizes to autophagosome membranes, a modification that allows LC3B-II to be distinguished from the soluble form LC3B-I [38,39]. Densitometric analysis of the LC3B-II band (14 kDa), normalized to GAPDH, is shown in Figure 4H,J. For TASK-3-depleted MDA-MB-231 cells, we actually observed a decrease in the lipid-conjugated form of LC3B (LC3B-II) protein compared to control cells (Figure 4H), while for TASK-3-depleted MCF-10F cells, we were unable to detect significant changes in the lipid-conjugated form of LC3B (LC3B-II) protein compared to control cells (Figure 4I,J). Taken together, these results are consistent with an autophagy-independent mechanism of proliferative impairment in both cell lines with reduced expression of TASK-3.

### 2.6. TASK-3 Knockdown in MDA-MB-231 and MCF-10F Cells Is Not Accompanied by an Increased Rate of Apoptosis

**Figure 5:**
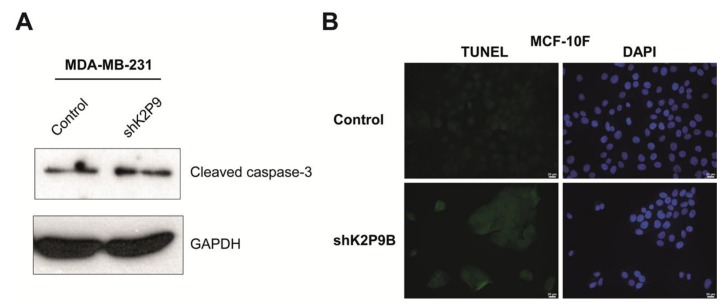
Assessment of apoptosis in cells with reduced expression of TASK-3. (**A**) Effect of knocking down TASK-3 on activation of caspase-3 in MDA-MB-231 cells. Representative immunoblots for the detection of cleaved caspase-3 and GAPDH in total cell lysates are shown; (**B**) effect of knocking down TASK-3 on apoptosis in MCF-10F cells assessed by TUNEL labeling. Representative fluorescence images of MCF-10F cell cultures transduced with the shRNA targeting TASK-3 (shK2P9B) or with a vector control (pMKO.1) are indicated. Cells were stained with DAPI (**blue**) and TUNEL-labeled (**green**).

Because TASK-3 has been previously associated with apoptotic cell death [40,41,42], we also explored the possibility that reducing the expression of TASK-3 might increase the apoptotic activity in both cell lines. To explore this possibility, the levels of cleaved caspase-3 were assessed by Western blotting in MDA-MB-231 cells (Figure 5A). The results indicated that knocking down TASK-3 in MDA-MB-231 cells did not lead to changes in the levels of cleaved (active) caspase-3 when compared to control cells (Figure 5A). Similarly, apoptosis induced by TASK-3 depletion was assessed by TUNEL assays and DAPI labelling in MCF-10F cells (Figure 5B). The results indicated that knocking down TASK-3 in MCF-10F cells did not lead to the appearance of pyknotic or fragmented nuclei visualized with DAPI staining, or the appearance of bright green fluorescent signal following TUNEL assay (Figure 5B). Altogether, these results indicate that apoptosis is not a factor contributing to the reduced proliferative capacity of TASK-3-depleted MDA-MB-231 and MCF-10F cells.

### 2.7. Analysis of the Cell Cycle Regulators

**Figure 6:**
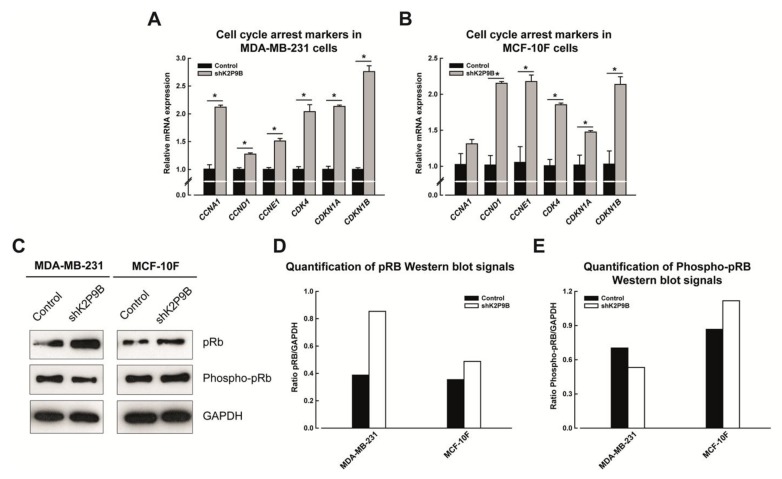
Analysis of cell cycle regulators in TASK-3-depleted MDA-MB-231 and MCF-10F cells. (**A**,**B**) The expression of the indicated cell cycle regulators was assessed by qPCR in MDA-MB-231 cells transduced with an shRNA targeting TASK-3 (shK2P9B) or vector control (pMKO.1); (**C**) representative immunoblots of pRB, Phospho-pRB and GAPDH, detected in total cell lysates, are shown. Quantitative densitometric analysis of blots are shown in (**D**,**E**), indicating the ratio between intensities of the pRB and Phospho-pRB bands of treated samples and the control sample, normalized on intensity of the GAPDH band. The values correspond to average ± SEM of three independent experiments. For (**A**,**B**) * *p* < 0.05 (*n* = 3 for each group, one-way ANOVA and Tukey HSD multiple comparison test post).

We next examined the expression of several cell cycle regulators involved in the G1/S cell cycle transition, in an attempt to explore the potential mechanisms involved in the implementation of senescence in TASK-3-deficient MDA-MB-231 cells, and cell cycle arrest observed in MCF-10F cells, following shRNA-mediated knockdown of TASK-3. To this end, mRNA expression of cell cycle regulators (*CCNA1*, *CCND1* and *CCNE1*, encoding cyclins A1, D1, and E1, respectively; *CDK4*, Cyclin-Dependent Kinase 4; *CDKN1A* and *CDKN1B*, Cyclin-Dependent Kinase Inhibitors 1A and 1B, also know as p21 and p27) was determined by qPCR (Figure 6A,B). The sequences of the specific primers used are listed in Table 1. Surprisingly, we observed a significant increase in the expression of genes encoding Cyclin A1 (*CCNA1*), Cyclin D1 (*CCND1*) and Cyclin E1 (*CCNE1*) following the knocking down of TASK-3 in MDA-MB-231 cells (Figure 6A). Similarly, a significant increase in the expression of genes encoding Cyclin D1 and Cyclin E1 in TASK-3-depleted MCF-10F cells was also observed (Figure 6B). However, these changes were also accompanied by a significant increase in the expression of cell cycle inhibitory genes p21 (*CDKN1A*) and p27 (*CDKN1B*) (Figure 6A,B). These results suggest that the induction of senescence that follows the reduced expression of TASK-3 in MDA-MB-231 cells might be preceded by a G1/S arrest. A similar cell cycle arrest might be involved in TASK-3-depleted MCF-10F cells, although this process is not followed by the implementation of a senescent phenotype. In line with the suppression of cell cycle progression, the levels of the retinoblastoma tumor suppressor protein (pRB), the main substrate of cyclin-dependent kinases, were altered in TASK-3-depleted cells (Figure 6C). We observed an increase in the total pRB, as well as a decrease of phospho-pRB, in response to TASK-3 knockdown in MDA-MB-231 cells when compared to control cells (Figure 6C). On the other hand, western blot analyses revealed an increase of the pRB as well as phospho-pRb in MCF-10F cells following TASK-3 knockdown (Figure 6C). These results indicate that shRNA for TASK-3 inhibits cell proliferation through a mechanism associated to activation of pRB.

## 3. Discussion

Human cancers are the result of a gradual and dynamic accumulation of genetic and epigenetic changes in somatic cells. These changes endow cancer cells with the ability to proliferate without control, invade surrounding tissues, and form colonies in distant parts of the organism. Efforts to systematize the cellular processes that are disrupted in cancer cells have produced a relatively short list of “hallmarks” of cancer [43]. Importantly, Ion channels are emerging as important modulators in the orchestration of at least some these hallmarks and, accordingly, they may now be considered as potential targets for the development of anti-cancer drugs [5]. However, it is presently difficult to assign a specific mechanism of action to a particular class of ion channel in the context of tumorigenesis.

The TASK-3 potassium channel is overexpressed in a variety of tumor cell lines and solid tumors from different histological origins, including breast, colon, lung and melanoma tissues [30,32,42,44,45]. However, the relative advantages for cancer cells to upregulate the expression of these channels and not others are far from clear.

Here, we investigated the role of TASK-3 channels in MDA-MB-231 human breast cancer cells and MCF-10F human mammary epithelial cells by first evaluating gene expression. We then tested the association of shRNA-mediated depletion of TASK-3 with senescence, autophagy, apoptosis and cell cycle arrest. We provide strong evidence for the quantitative mRNA transcript detection and protein immunolocalization of TASK-3 channels in both cell lines. The Immunofluorescence characterization of TASK-3 channels revealed a staining pattern that was consistent with membrane localization. These results indicate that TASK-3 channel is stably expressed on the cell surface of the MDA-MB-231 and MCF-10F cells, and are in agreement with what was previously reported [44].

In order to examine the effects of TASK-3 deficiency in MDA-MB-231 and MCF-10F cells, we designed shRNA constructs that effectively reduced the expression of this channel. Importantly, the expression of the highly homologous TASK-1 channel was not affected, suggesting that the shRNA-mediated knockdown of TASK-3 was specific. We also conducted western blot analyses and functional evaluation (macroscopic outward K^+^ currents) for TASK-3 channels in order to confirm that the protein was reduced MDA-MB-231 and MCF-10F cells. Our results corroborate the effectivity and specificity for the knockdown of TASK-3.

The possibility of a role of TASK-3 channels in the proliferation of MDA-MB-231 and MCF-10F cells was also explored. In keeping with other reports [46], the proliferative ability of cells deficient in TASK-3 was greatly impaired. Therefore, based on our cell proliferation experiments, we can conclude that knocking down TASK-3 might cause a strong reduction in cell viability.

To elucidate the mechanism that may explain the inhibition of proliferation observed in TASK-3-depleted cells, senescence, autophagy, apoptosis and cell cycle arrest were examined. The first marker used for the identification of senescent cells was senescence-associated β-galactosidase (SA-β-gal) staining [47]. We showed that this marker was only significantly increased in TASK-3-depleted MDA-MB-231 cells (Figure 4A–C). Autophagy, a plausible mechanism of cell death investigated, was also unlikely to contribute to the proliferative arrest of these cells (Figure 4G–J).

Reduced expression of the TASK-3 channel has been associated with apoptotic cell death [40,41,42]. Nonetheless, in our hands, the TASK-3 knockdown has no significant effect over apoptotic rates (Figure 5A,B). This discrepancy can be explained by differences in the cellular phenotype displayed by cancerous cells versus non-tumorigenic cells such as MCF-10F cells. Of note, MDA-MB-231 cells have very high levels of phospholipase D (PLD) activity [48] relative to other breast cancer cells, providing a survival signal that suppresses apoptosis when these cells are subjected to apoptotic stress [48]. Also, MDA-MB-231 cells have shown resistance to genotoxic drugs, such as etoposide and cisplatin, chemotherapeutic agents that activate the mitochondrial apoptotic pathway [49]. Taken together, senescence induction in MDA-MB-231 cells was independent of autophagy and apoptosis, ruling out these mechanisms as the main contributors of the cell proliferation impairment observed in TASK-3-depleted cells.

Surprisingly, TASK-3-deficient MDA-MB-231 and MCF-10F cells showed a significant increase in the expression of cell cycle promoting cyclins (encoding Cyclin D1 and Cyclin E1), which was, however, accompanied by an increase in the expression of the CDK inhibitors p21 and p27 [50,51] (Figure 6A,B).

CDK inhibitors (CKI) play a crucial role in cell cycle arrest. CKIs bind either CDK or CDK/Cyclin complexes to inhibit CDK activity and cause cell cycle arrest [52]. In particular, p21 and p27, which belong to the Cip/Kip family of CKIs, cause cell cycle arrest specifically by inactivating the CDK2/Cyclin E complexes in the G1 phase. In addition, p21 has also been shown to inhibit DNA synthesis [52,53].

The canonical model of the G1-to-S cell cycle transition involves a CDK4/Cyclin D-dependent initial phosphorylation of pRB and the consequent release of E2F transcription factors [54]. In turn, E2F factors promote transcription of Cyclin E, leading to activation of CDK2/Cyclin E complexes, which further phosphorylates pRB and release more E2F, thus providing a positive feedback loop. In line with this model, TASK-3 depletion in MDA-MB-231 cells seems to affect the kinase activity of CDK-containing complexes, reflected in a reduction in the levels of phosphorylated pRB, at least in MDA-MB-231 cells (Figure 6C). Therefore, it is plausible that inhibition of Cyclin/CDK complexes secondary to upregulation p21 and p27 contributes to orchestrate cell cycle arrest and senescence in MDA-MB-231 cells in the context of TASK-3 depletion.

On the other hand, TASK-3-depletion in MCF-10F cells did not have a significant effect on phosphorylation of pRB (Figure 6C). Although both qPCR and Western blot analyses show an increase in the expression of the cell cycle drivers Cyclin D1, Cyclin E1 and pRB, the concomitant increase in the expression of the CKIs p21 and p27 may explain the cell cycle arrest observed during the G1/S phase, although it does not seem to involve changes in phosphorylation of pRB. However, increases in gene expression of CKIs support the finding that TASK-3 knockdown induces cell cycle arrest in MCF-10F human mammary epithelial cells via the inhibition of the activity of CDK/Cyclin complexes.

Our current results indicate that down-regulation of TASK-3 expression, or prevention of TASK-3 up-regulation, reduced the proliferation rate of the human mammary epithelial MCF-10F cells. In this study, we found that prevention of TASK-3 up-regulation using shRNA-mediated gene silencing indeed potentiates the growth inhibitory effects in MCF-10F cells. Thus, these results confirm the previously described finding that TASK-3 regulates proliferative advantages in breast cancer cell lines [24,55].

In summary, in this work we corroborate the presence and localization patterns of TASK-3 channels in MDA-MB-231 and MCF-10F cells, by means of gene expression analysis by quantitative real-time PCR, Western blotting and immunofluorescence. In addition, TASK-3 knockdown exerted an inhibitory effect on the proliferation rate of both cell lines, generated by cell cycle arrest mediated by CDK inhibitor upregulation.

## 4. Materials and Methods

### 4.1. Cell Culture and Reagents

The human MDA-MB-231, MCF-10F and HEK-293T cell lines were obtained from the American Type Culture Collection. MDA-MB-231 cells were propagated in RPMI-1640 medium (HyClone; GE Healthcare Life Sciences, Logan, UT, USA) supplemented with 10% fetal bovine serum (FBS), 10 µg/mL insulin. MCF-10F cells were maintained in 1:1 DMEM/F-12 nutrient mixture supplemented with 5% horse serum, 10 µg/mL insulin, 10 ng/mL epidermal grown factor (EGF), 10 mg/mL hydrocortisone, all medias were maintained with 25 mg/mL gentamycin and 250 ng/mL amphotericin B. Cells were maintained at 37 °C in a humidified atmosphere containing 5% CO_2_.

### 4.2. Immunocytochemistry

Cellular localization of TASK-3 in MDA-MB-231 and MCF-10F cells was analyzed by immunofluorescence as described previously [16]. Briefly, cells were seeded on coverslips, fixed in 4% paraformaldehyde (PFA)/1× phosphate-buffered saline (PBS) for 20 min at room temperature, and permeabilized with 2% bovine serum albumin in 1× PBS containing 0.1% Triton X-100 for 30 min. After incubating in blocking buffer, cells were incubated with anti-TASK-3 antibody (1:100; sc-11317, Santa Cruz Biotechnology, USA) overnight at 4 °C. Negative controls were treated in the same way, replacing the primary antibody with 1× PBS. Cells were then incubated with an Alexa Fluor 594-conjugated secondary antibody (ab150132; Abcam, Cambridge, MA, USA) at 1:1000 dilution, for 1 h at room temperature. Finally, DAPI (4′,6-diamidino-2-phenylindole, 0.1 µg/mL for 5 min) was used to stain cell nuclei. Immunofluorescence images were acquired in a fluorescence microscope (Olympus BX53; Center Valley, PA, USA), coupled to a CCD camera. Digital images were acquired using the Q-Capture Pro 7 software (QImagine, Surrey, Canada). Each staining was done in triplicate in 3 independent experiments.

### 4.3. TASK-3 Silencing with Short Hairpin RNA (shRNA)

In order to generate retroviral vectors expressing short hairpin RNAs (shRNAs) targeting TASK-3, the following oligodeoxyribonucleotide sequences were annealed and subcloned between the AgeI and EcoRI restriction sites of pMKO.1-puro vector: shK2P9A (sense, 5′-CCG GGC TGA AGC GCA TTA AGA AG-3′ and antisense, 5′-AAT TCA AAA AGC TGA AGC GCA TTA AGA AG-3′); shK2P9B (sense, 5′-CCG GGC TTC ATC ACG TTG ACT AC-3′ and antisense, 5′-AAT TCA AAA AGC TTC ATC ACG TTG ACT AC-3′), and shK2P9C (sense, 5′-CCG GCC ATG AAC AGT GAG GAT GA-3′ and antisense, 5′-AAT TCA AAA ACC ATG AAC AGT GAG GAT GA-3′). Retroviral particles were produced by transfection (Lipofectamine 2000; Invitrogen Life Technologies, Carlsbad, CA, USA) of HEK-293T cells with pMKO.1 constructs and the appropriate packaging plasmids. MDA-MB-231 and MCF-10F cells were cultured in 6-well plates until 50–60% confluence. Cells were then transduced with shRNA or the pMKO.1-puro empty vector (control), and selected in Puromycin (10 µg/mL)-containing medium for 3–4 days until a stable selection. The knockdown of TASK-3 was evaluated at different times to check the silencing.

### 4.4. Patch-Clamp Recording

Standard whole cell patch-clamp recordings were performed as described elsewhere [56], using a PC-501A amplifier (Warner Instruments, Hamden, CT, USA). The voltage pulse generator and analysis programs were from Axon Instruments. Patch-clamp pipettes had resistances of 3–5 MΩ. The pipette solution contained (mM): 140 KCl, 0.5 CaCl_2_, 5 EGTA, 10 Hepes, 2 K_2_-ATP, 1 MgCl_2_; pH was adjusted to 7.3 with KOH. The bath solution contained (mM): 120 NaCl, 4 KCl, 2 MgCl_2_, 0.5 CaCl_2_, 10 glucose, 10 Hepes; pH 5.0, 7.4, and 8.0 were adjusted with NaOH. Cells were held at −80 mV. Then, step pulses of 500 ms of durations were applied from −100 to +80 mV, followed by a step to −80 mV.

### 4.5. Trypan Blue Exclusion

Cell viability was assessed using the Trypan blue exclusion assay. MDA-MB-231 and MCF-10F cells undergoing shRNA-mediated knockdown and growing in six-well tissue culture plates were treated for 48 h with DMEM containing 0.1% ethanol. The cells were then harvested by trypsinization and centrifugation at 300× *g* for 5 min. Pellets were resuspended in 0.4% Trypan blue solution (Sigma-Aldrich, St. Louis, MO, USA), and live (unstained) and dead (stained blue) cells were counted using a hemocytometer to determine the total number of viable cells. The percentage of surviving cells was calculated based on the ratio of viable cell to total cell population from each well. The proliferation rate was calculated based on the number of viable MDA-MB-231 or MCF-10F cells infected with vector control (pMKO.1 puro) versus MDA-MB-231 or MCF-10F cells infected with shRNAs against K2P9 (shK2P9A, shK2P9B, and shK2P9C). Experiments were repeated to confirm the accuracy of the results.

### 4.6. Extraction and Quantification of mRNA by Real Time PCR

Total RNA was extracted from MDA-MB-231 and MCF-10F cells using TRIzol Reagent (Life Technologies) followed by an additional DNase treatment (TURBO DNA-free kit; Life Technologies). First-strand cDNA was primed with oligo(dT) from 1 μg of RNA and synthesized using the RevertAid H Minus First Strand cDNA Synthesis Kit (Thermo Fisher Scientific, Waltham, MA, USA) at 42 °C for 60 min. The cDNA generated was used as a template for PCR amplification using specific primers (Table 1).

Real-time PCR reactions consisted of 5 μL of 2× Maxima SYBR Green/ROX qPCR Master Mix (Thermo Fisher), 250 nM of each primer, and 100 ng of cDNA template. The cycling conditions were the following: a cycle of 95 °C for 10 min, followed by 40 cycles at 95 °C for 15 s, 60 °C for 15 s, 72 °C for 20 s, a final cycle at 95 °C for 1 min, and a melting curve from 55 °C to 95 °C at 0.5 °C/s increments. These assays were performed in triplicate in a Stratagene Mx3000P real-time thermal cycler, and analyzed with MxPro qPCR software (Agilent Technologies, Santa Clara, CA, USA). All pairs of primers were tested and the efficacies evaluated (only those giving efficiencies of 90–100% were selected). Additionally, gel electrophoresis and melting curve analyses, were performed in order to confirm the specificities of the PCR products. The expression of each gene was normalized to ribosomal protein L19 (RPL19).

### 4.7. Protein Extraction and Western Blotting

Cultured MDA-MB-231 and MCF-10F cells were gently scraped, pelleted by centrifugation at 5000× *g* for 5 min at 4 °C, resuspended and lysed in ice-cold RIPA buffer supplemented with protease and phosphatase inhibitors. For Western blot, 60 μg of protein were subjected to 10% SDS-polyacrylamide electrophoresis. Proteins were then transferred to nitrocellulose membranes (Thermo Fisher) and incubated with primary antibodies against TASK-3 (1:1000 antibody dilutions).

Membranes were then incubated with 1:5000 peroxidase-conjugated secondary antibodies and antibody-antigen complexes were then visualized using ECL Plus Kit and a hyper film MP (GE Healthcare, Little Chalfont, UK). Quantification of protein levels in the Western blots was performed using ImageJ software version 1.48d (National Institutes of Health, Bethesda, MD, USA). The following antibodies were used in this study: goat anti-TASK-3 (sc-11317; Santa Cruz Biotechnology, Santa Cruz, CA, USA), anti-LC3B (D11 XP rabbit mAb; Cell Signaling Technology, USA), anti-cleaved caspase-3 (Asp175 5A1E rabbit mAb; Cell Signaling Technology, Danvers, MA, USA), anti-Rb (D20 rabbit mAb; Cell Signaling), anti-Phospho-Rb (D59B7 rabbit mAb; Cell Signaling), mouse anti-GAPDH (sc-365062; Santa Cruz) and a secondary rabbit anti-goat HRP (sc-2768; Santa Cruz).

### 4.8. Senescence-Associated β-Galactosidase Staining Assay

Detection of senescence-associated β-galactosidase activity (SA-β-gal) was carried out essentially as described before [47]. Briefly, cultured cells were washed once in 1× PBS (pH 7.4) and fixed with 2% PFA/0.2% glutaraldehyde for 10–15 min at room temperature. Cells were then washed twice with 1× PBS and stained with fresh SA-β-galactosidase staining solution (1 mg/mL 5-bromo-4-chloro-3-indolyl-β-d-galactopyranoside, 40 mM citric acid/sodium phosphate buffer (pH 6.0), 5 mM potassium ferrocyanide (K_3_Fe[CN]_6_), 5 mM potassium ferricyanide (K_4_Fe[CN]_6_), 150 mM NaCl, and 2 mM MgCl_2_) overnight at 37 °C. Following this incubation, cells were washed twice in 1× PBS, and examined with a BX53 fluorescence microscope (Olympus), coupled to a CCD camera. Digital images were taken using Q-Capture Pro 7 software (QImagine). The images were analyzed using the software BioImageXD 1.0 (Free Software Foundation) and the stained cells counted. All these experiments were performed 3 times by using independent cultures.

### 4.9. Assessment of Apoptosis

Apoptosis in adherent cells was assessed by terminal deoxynucleotidyl transferase-mediated DNA nick-end labelling (TUNEL) assays. For nuclear staining purposes, cells were fixed with freshly prepared 4% PFA and incubated in a DAPI solution (0.1 µg/mL during 5 min). For TUNEL labelling, the DeadEnd Fluorometric TUNEL system (Promega) was used according to the manufacturer’s instructions. Cells were analyzed by fluorescence microscopy and images were digitally acquired as indicated above.

### 4.10. Statistical Analysis

Data were compiled and analyzed with the SigmaPlot software version 12.0 (Systat Software, San Jose, CA, USA). Group differences were calculated with one- or two-way ANOVA with post-hoc Tukey HSD test. *p* < 0.05 was considered statistically significant and all data shown are mean ± standard error of mean (SEM).

## 5. Conclusions

We corroborate the overexpression and functional status of TASK-3 in the MDA-MB-231 human breast cancer cell line and also in the non-transformed human breast cancer cell line MCF-10F. In addition, we show that the TASK-3-silencing in MDA-MB-231 cells suppresses cell proliferation by inducing senescence. To MCF-10F cells, TASK-3 knockdown exerted an inhibitory effect on the proliferation rate, generated by cell cycle arrest mediated by CDK inhibitor upregulation. This anti-proliferative effect is likely mediated by p21 or p27. Altogether, our results have confirmed the role of TASK-3 in proliferation and highlight this channel as a potential target for the development of specific inhibitors that can be used in the treatment of triple-negative breast cancers.

## Figures and Tables

**Table 1:** Primer sets used for quantitative real time RT-PCR analysis.

Gene	Common Name	GenBank Accession	Abbreviation	Primer Pair, Sense (5′–3′)	Product Size (bp)
*KCNK9*	K2P9, TASK-3	NM_001282534.1	qHsKCNK9_F1	5′-GTC TCA TTT TCC CCC ACC TTT CCA G-3′	148
			qHsKCNK9_R1	5′-GGG TGG GGT GAG AAA TGT AAG GCA-3′	
*KCNK3*	K2P3, TASK-1	NM_002246.2	qHnP3_F	5′-GGT GCT CAT CGG CTT CTT CT-3′	199
			qHnP3_R	5′-GAA GCT GAA GGC CAC GTA CT-3′	
*CCNA1*	Cyclin A1	NM_003914.3	qHsCCNA1_F	5′-TGA AAT AAG GCA CAG ACC CAA AGC A-3′	89
			qHsCCNA1_R	5′-ACC AGC CAG TCC ACC AGA ATC GT-3′	
*CCND1*	Cyclin D1	NM_053056.2	qHsCCND1_F	5′-GCT CCT GTG CTG CGA AGT GGA A-3′	126
			qHsCCND1_R	5′-TTT GAA GTA GGA CAC CGA GGG CG-3′	
*CCNE1*	Cyclin E1	NM_001238.2	qHsCCNE1_F	5′-AAG GTT TCA GGG TAT CAG TGG TGC G-3′	191
			qHsCCNE1_R	5′-GGC TTT CTT TGC TCG GGC TTT G-3′	
*CDK4*	CDK4	NM_000075.3	qHsCDK4_F	5′-TCG TGA GGT GGC TTT ACT GAG GCG-3′	194
			qHsCDK4_R	5′-TCC TTG ATC GTT TCG GCT GGC A-3′	
*CDKN1A*	p21, Cip1	NM_001291549.1	qHsCDKN1A_F	5′-TGT CCG TCA GAA CCC ATG C-3′	139
			qHsCDKN1A_R	5′-AAA GTC GAA GTT CCA TCG CTC-3′	
*CDKN1B*	p27, Kip1	NM_004064.4	qHsCDKN1B_F	5′-GGG TCT GTG TCT TTT GGC TCC GA-3′	94
			qHsCDKN1B_R	5′-CCG CCT CTC TCG CAC TCT CAA A-3′	
*RPL19*	L19	NM_000981	qHsRPL19_F	5′-CAT CCG CAA GCC TGT GAC G-3′	132
			qHsRPL19_R	5′-TGT GAC CTT CTC TGG CAT TCG-3′	

K2P, two-pore domain potassium channels; TASK-1 and TASK-3, TWIK-related acid-sensitive K^+^ channels 1 and 3; CCNA1, CCND1 and CCNE1, Cyclins A1, D1, and E1; CDK4, Cyclin-Dependent Kinase 4; *CDKN1A* and *CDKN1B*, Cyclin-Dependent Kinase Inhibitors 1A and 1B; RPL19, ribosomal protein L19.

## Supplementary Materials

Supplementary materials can be found at http://www.mdpi.com/1422-0067/19/4/1033/s1.
