# Supplementary material for: TASK-3 Downregulation Triggers Cellular Senescence and Growth Inhibition in Breast Cancer Cell Lines
Source: Int J Mol Sci. 2018 Mar 29;19(4):1033. doi: 10.3390/ijms19041033 (PMC5979529; doi:10.3390/ijms19041033)
Supplement: Supplementary file 1 [file ijms-19-01033-s001.pdf]

Supplementary figures

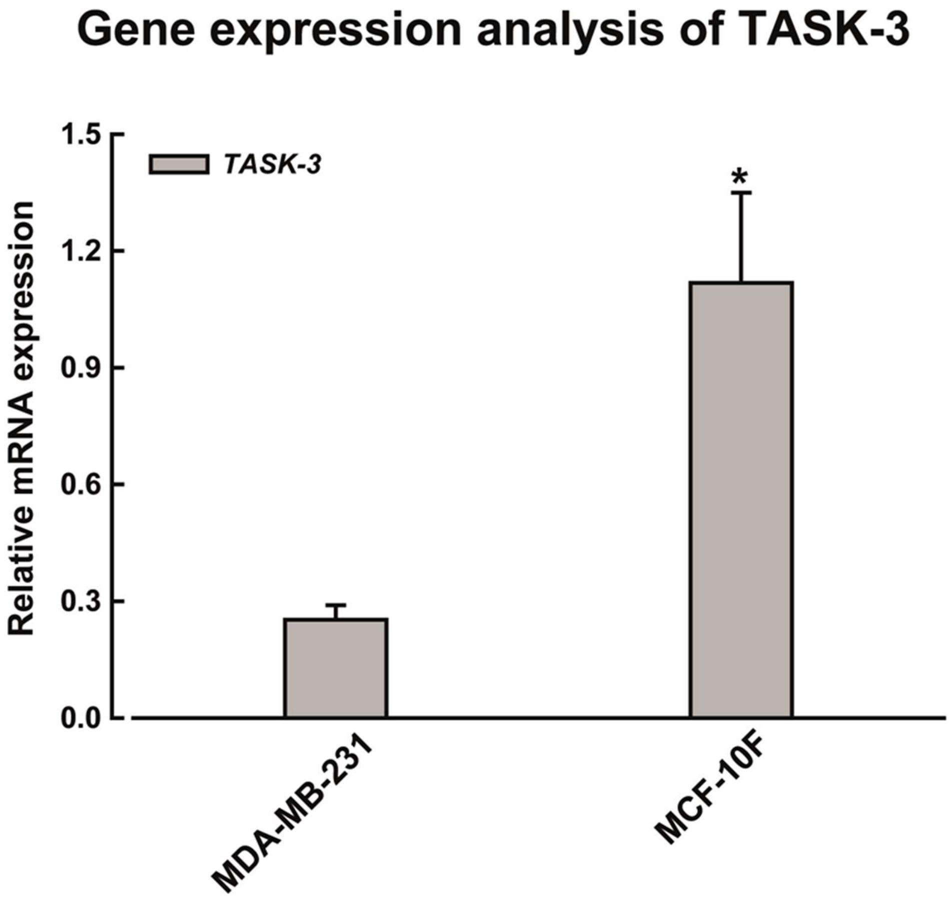

Figure S1. Gene expression analysis of TASK-3 in MDA-MB-231 and MCF-10F cells. The expression of TASK-3 (*KCNK9*) was assessed by RT-qPCR. Gene expression was normalized against *RPL19*. Data correspond to means ± SEM of three independent experiments. *: $P < 0.05$, between the cell lines, based on one-way ANOVA with Tukey HSD post-test.

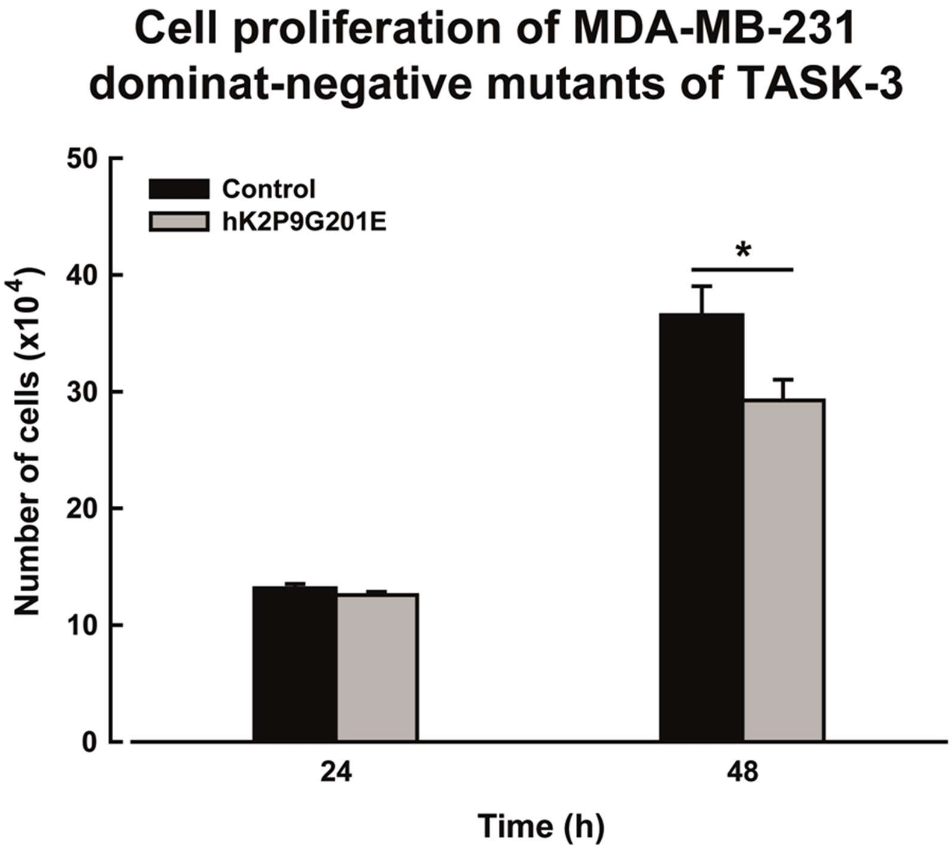

Figure S2. Effects of the dominant-negative form of TASK-3 (hK2P9G201E) in proliferation of MDA-MB-231 cells. Cell proliferation assays were performed on MDA-MB-231 cells following transfection with either vector control (pMax) or hK2P9G201E. The values correspond to average ± SEM of four independent experiments. *: $P < 0.05$, compared with the control, based on one-way ANOVA with Tukey HSD post-test.

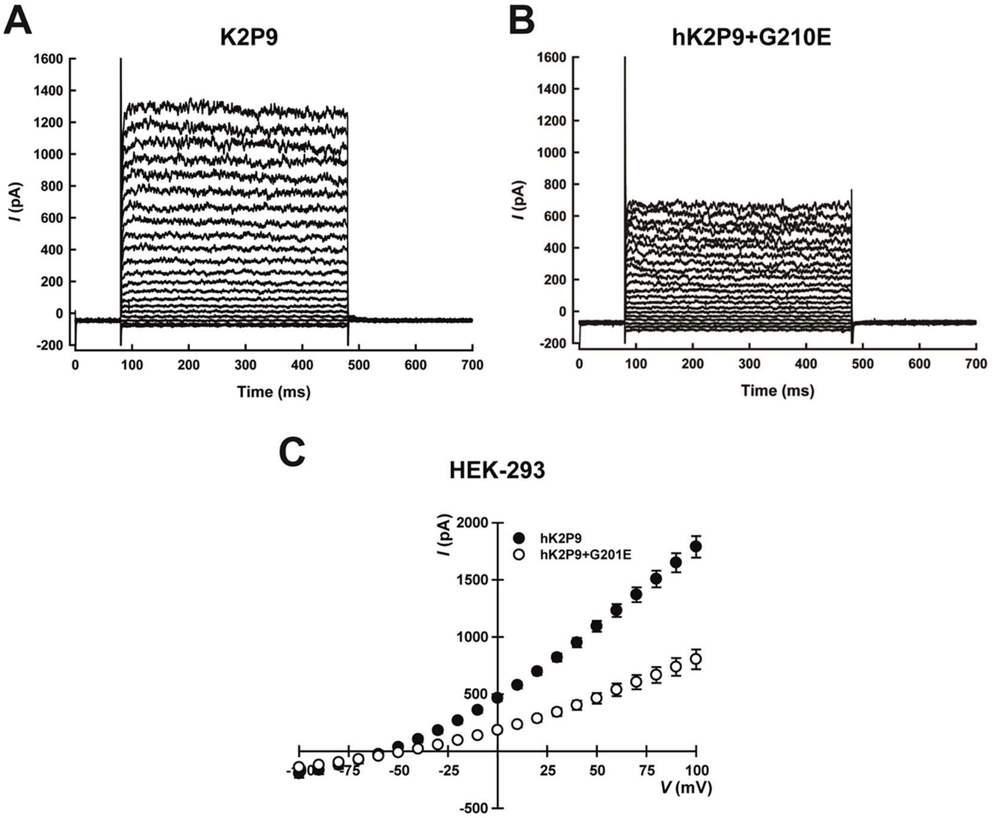

Figure S3. Whole-cell patch clamp measurements in cells transfected with the dominant-negative variant of TASK-3 (hK2P9G201E). (**A,B**) Representative current traces of TASK-3 (hK2P9), recorded in HEK-293 cells after co-trasfection of hK2P9+empty vector (pMax, as a control) or hK2P9+G201E. (**C**) Current-voltage relationships determined in both treatments are shown.
